# Supplementary material for: Nutritional compensation of the circadian clock is a conserved process influenced by gene expression regulation and mRNA stability
Source: PLoS Biol. 2023 Jan 5;21(1):e3001961. doi: 10.1371/journal.pbio.3001961 (PMC9848017; doi:10.1371/journal.pbio.3001961)
Supplement: S1 Raw Images — All immunoblots are included with 1 or 2 TIF images each showing raw blots uncropped and labeled with antibody used, method, sample ID, and molecular weight markers (when applicable). “X”s mark lanes and samples that were not shown in Fig 3D. (PDF) [file pbio.3001961.s028.pdf]

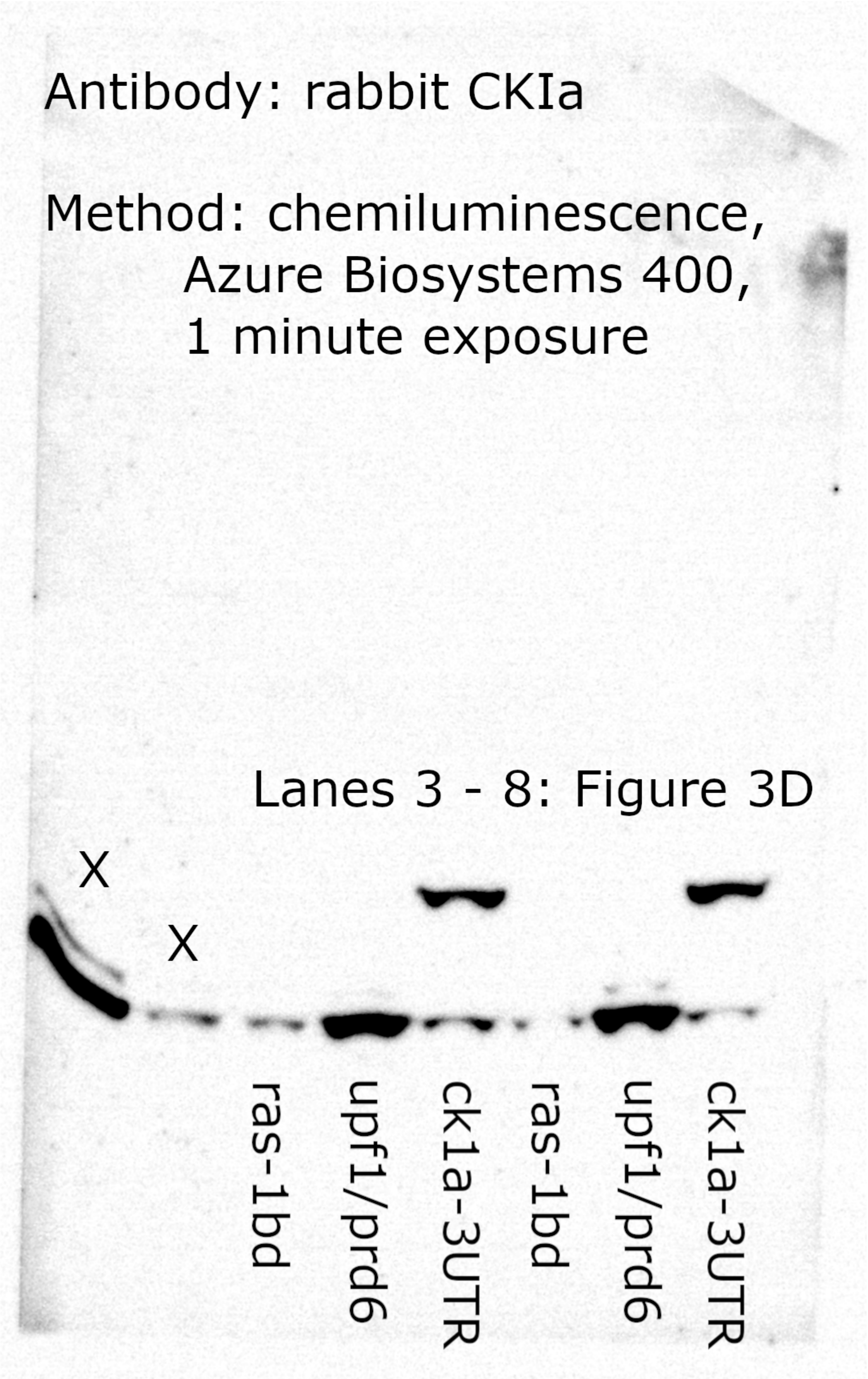
Antibody: rabbit CKIa
Method: chemiluminescence,
Azure Biosystems 400,
1 minute exposure
Lanes 3 - 8: Figure 3D
X
X
ras-1bd
upf1/prd6
ck1a-3UTR
ras-1bd
upf1/prd6
ck1a-3UTR

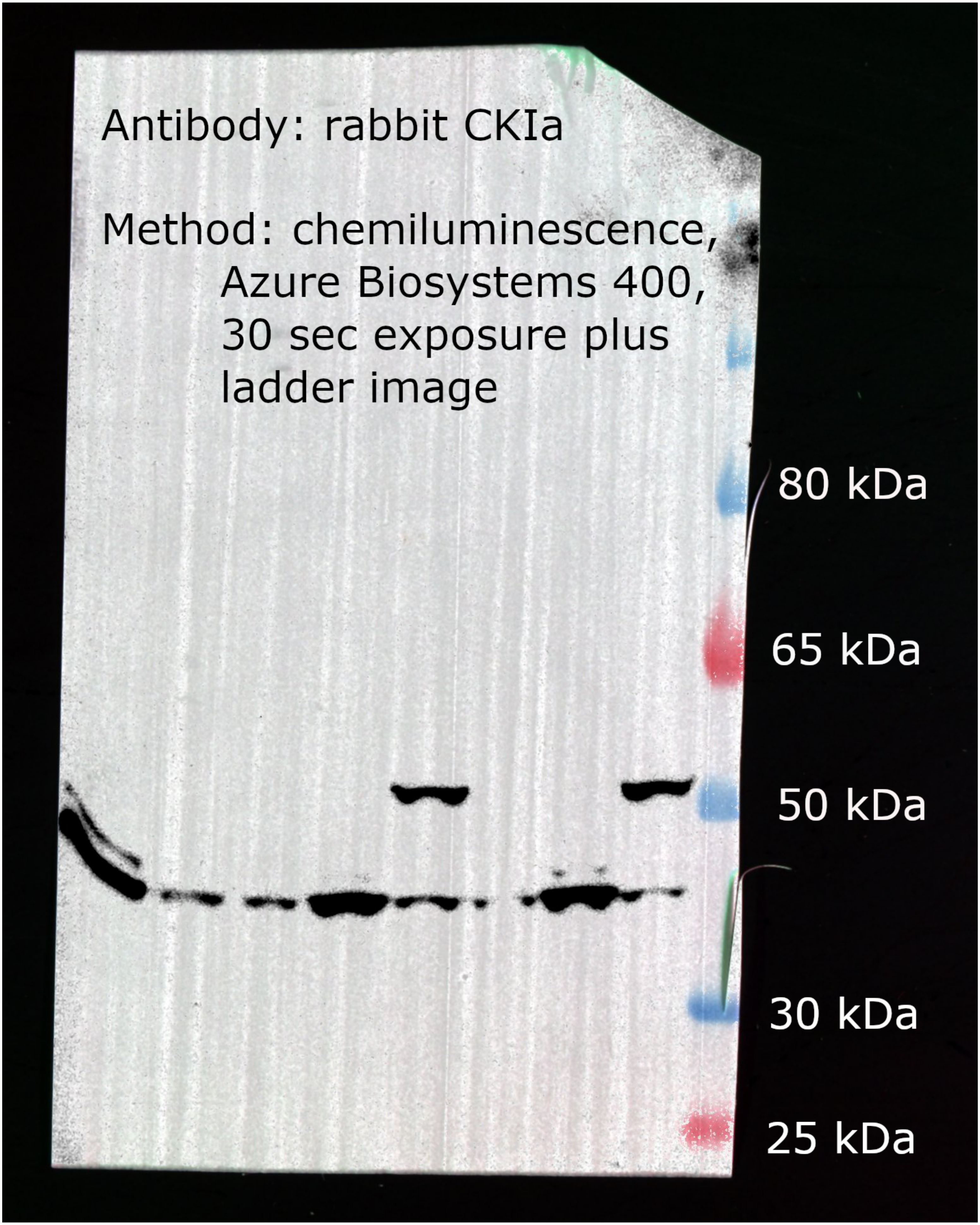
Antibody: rabbit CKIa
Method: chemiluminescence,
Azure Biosystems 400,
30 sec exposure plus
ladder image
80 kDa
65 kDa
50 kDa
30 kDa
25 kDa

Antibody: mouse a-tubulin

Method: chemiluminescence,
Azure Biosystems 400,
3 minute exposure

Lanes 1 - 6: Figure 3D X X

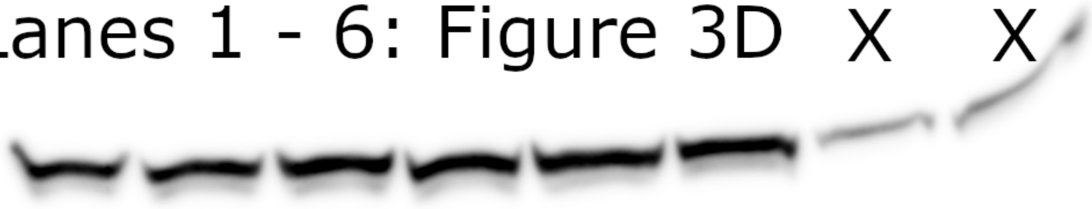

ras-1bd

upf1/prd6

ck1a-3UTR

ras-1bd

upf1/prd6

ck1a-3UTR

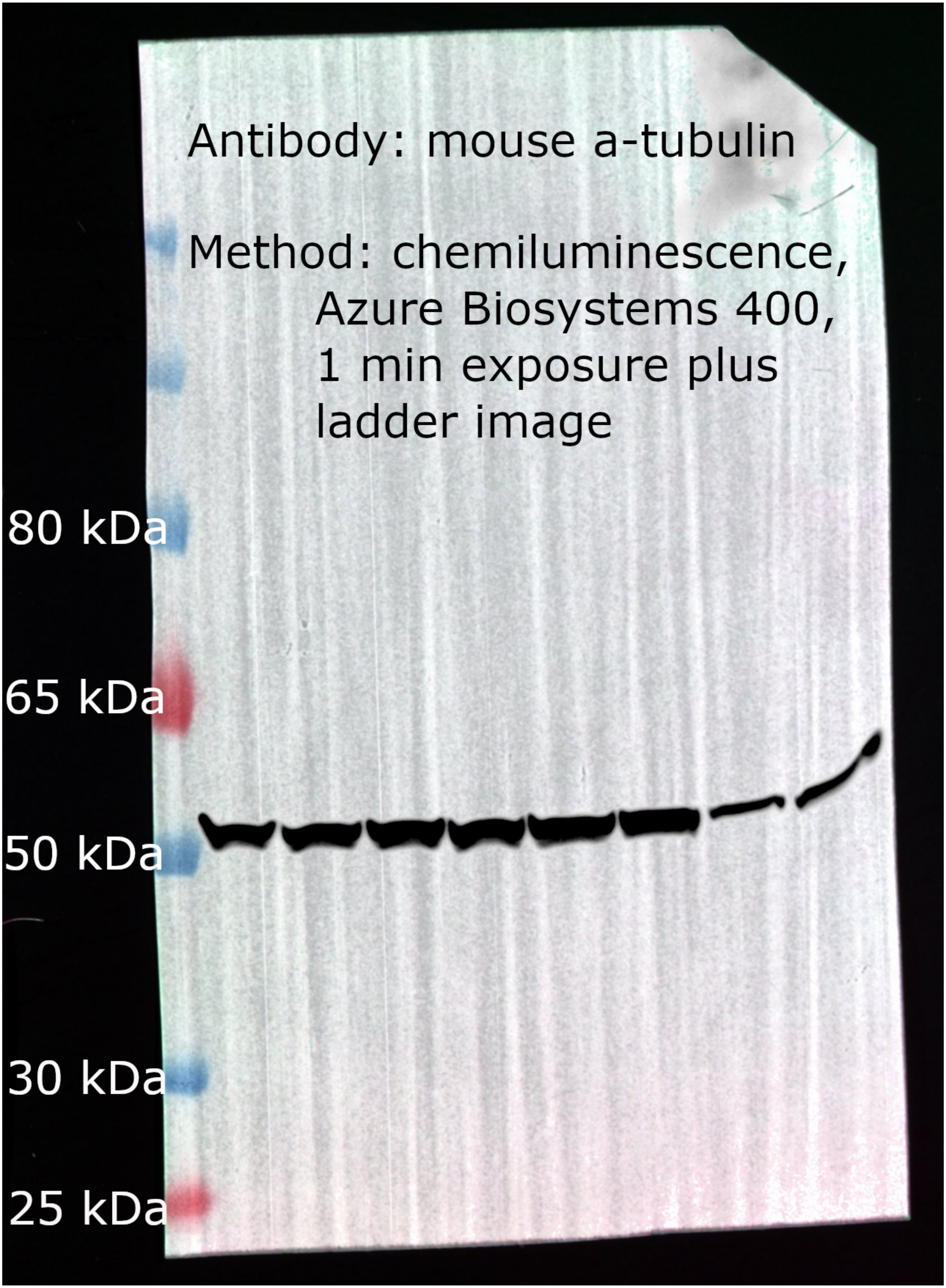
Antibody: mouse a-tubulin
Method: chemiluminescence, Azure Biosystems 400, 1 min exposure plus ladder image
80 kDa
65 kDa
50 kDa
30 kDa
25 kDa

Antibody: rabbit WC-2

Method: chemi,
Azure 400,
3 min exposure
Lane 1 - 4: Figure 3D

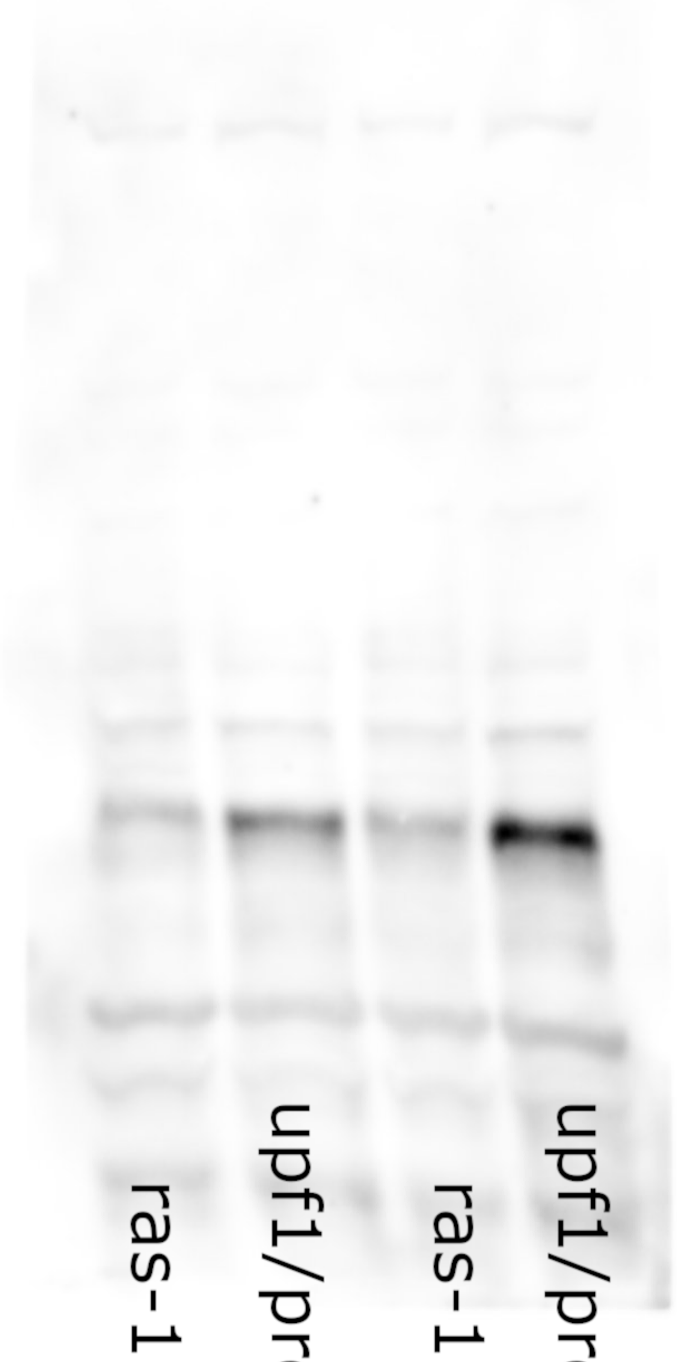

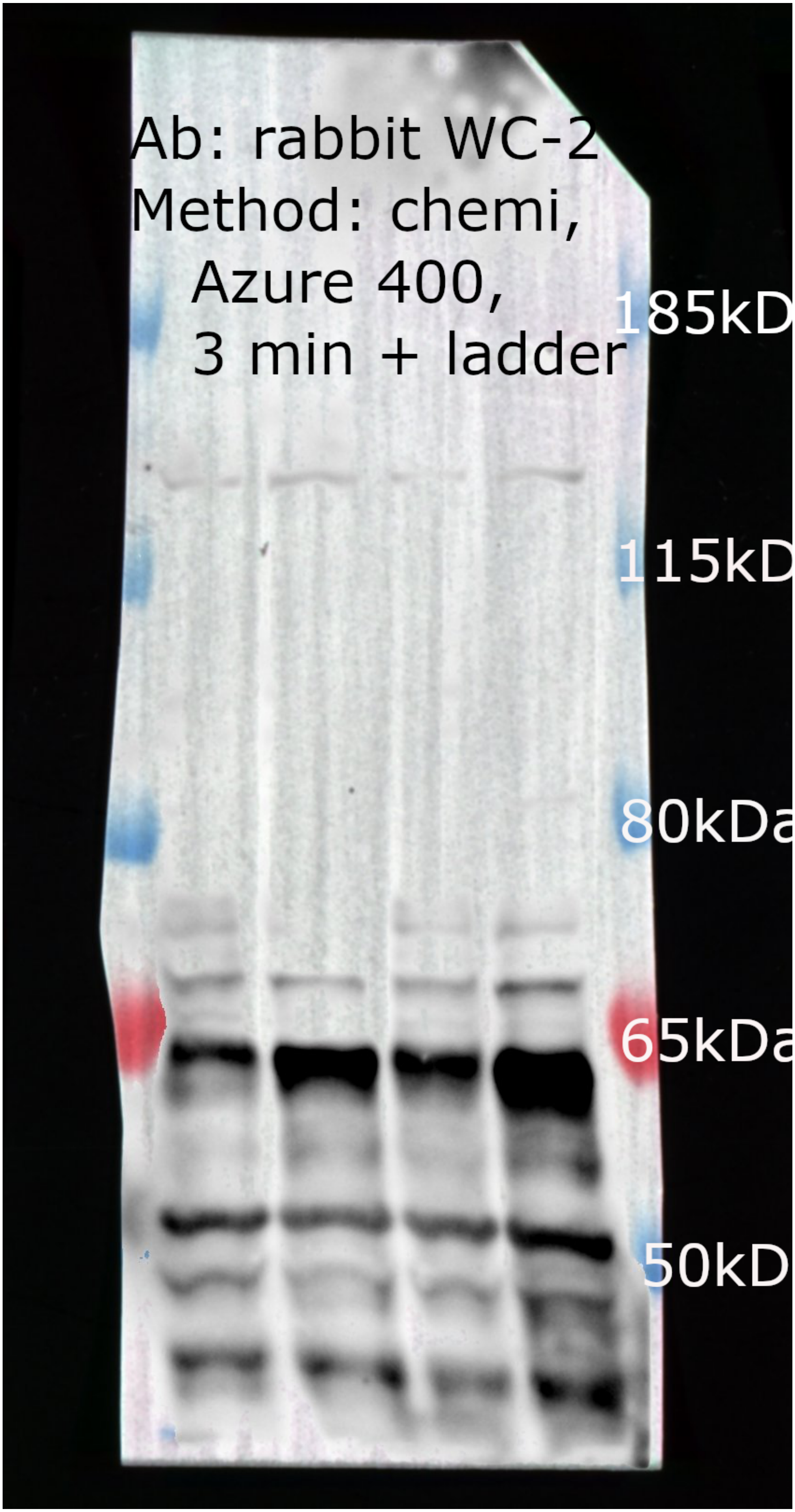
Ab: rabbit WC-2
Method: chemi,
Azure 400,
3 min + ladder
185kD
115kD
80kDa
65kDa
50kD

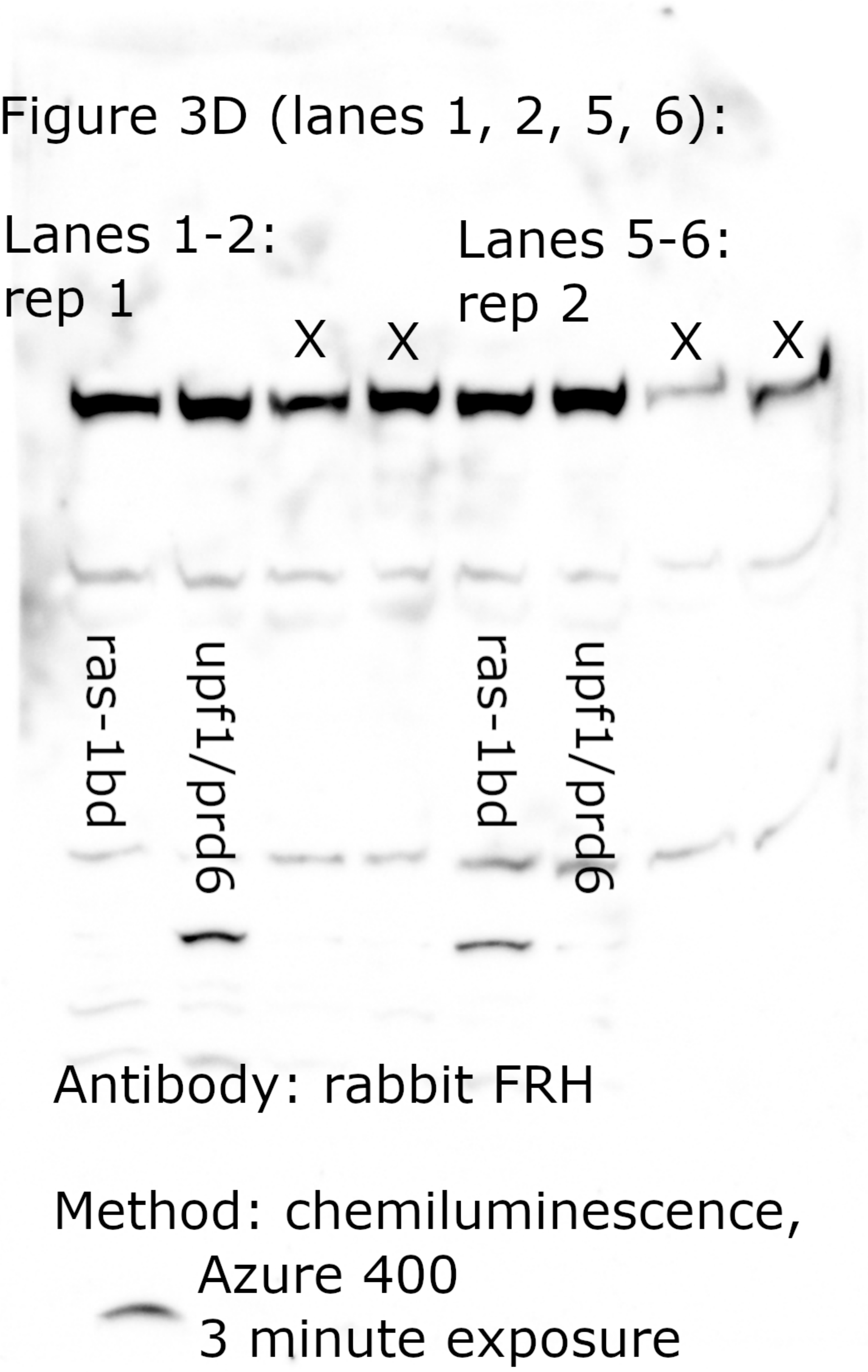
Figure 3D (lanes 1, 2, 5, 6):
Lanes 1-2:
rep 1
Lanes 5-6:
rep 2
X
X
X
X
ras-1bd
upf1/prd6
ras-1bd
upf1/prd6
Antibody: rabbit FRH
Method: chemiluminescence,
Azure 400
3 minute exposure

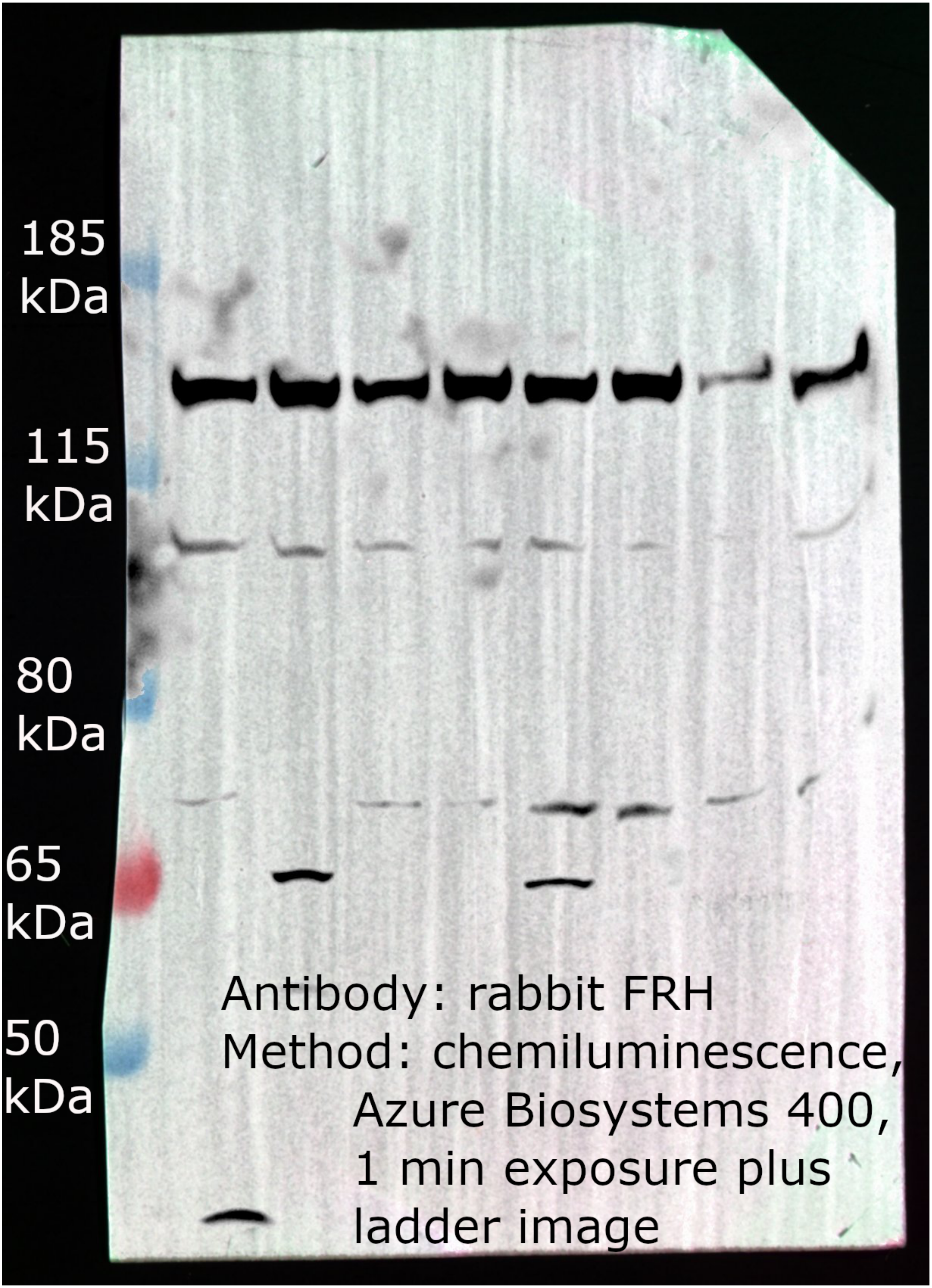
185 kDa
115 kDa
80 kDa
65 kDa
50 kDa
Antibody: rabbit FRH
Method: chemiluminescence,
Azure Biosystems 400,
1 min exposure plus
ladder image

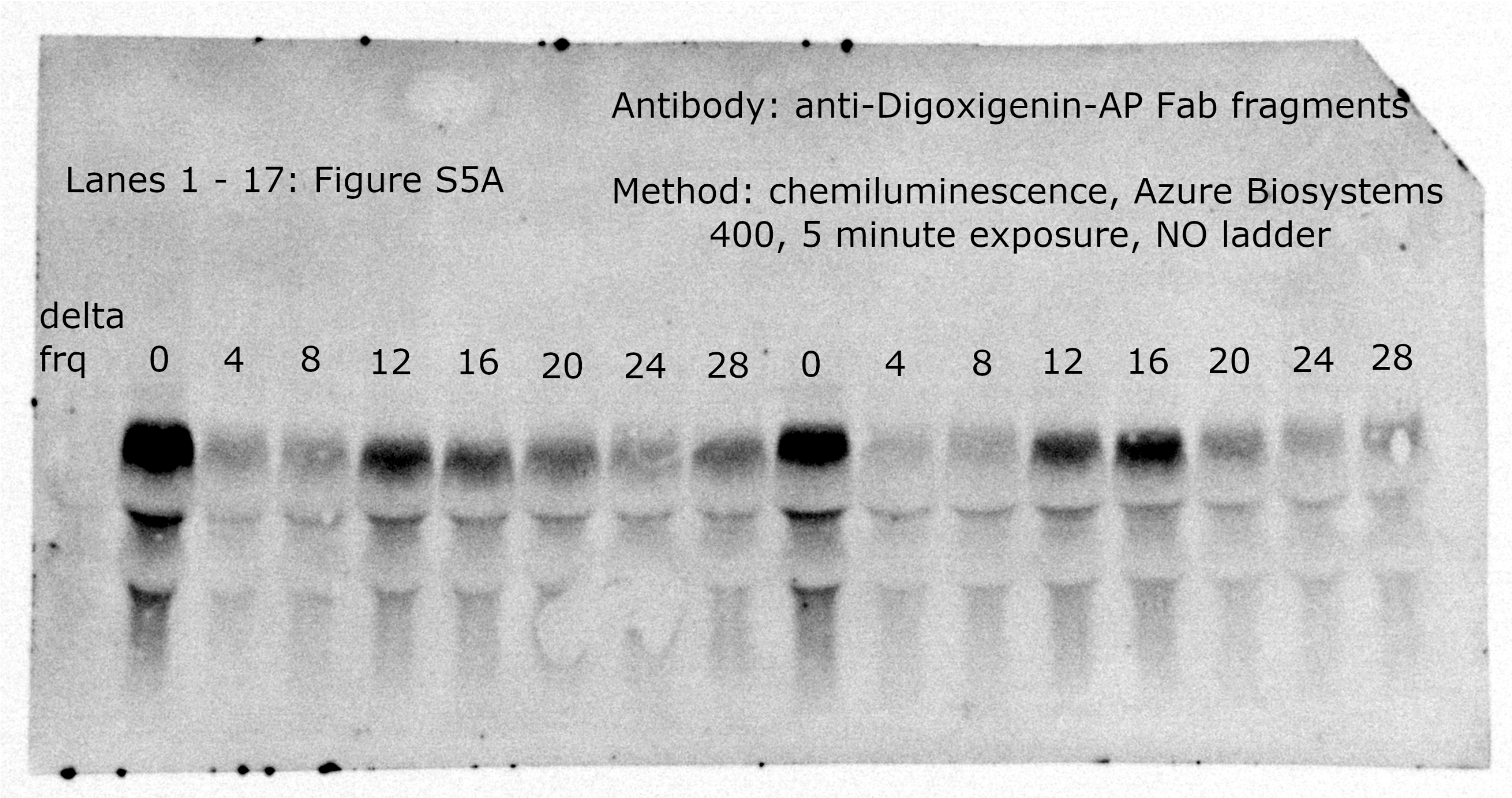
Antibody: anti-Digoxigenin-AP Fab fragments
Lanes 1 - 17: Figure S5A
Method: chemiluminescence, Azure Biosystems 400, 5 minute exposure, NO ladder
delta frq
0 4 8 12 16 20 24 28 0 4 8 12 16 20 24 28

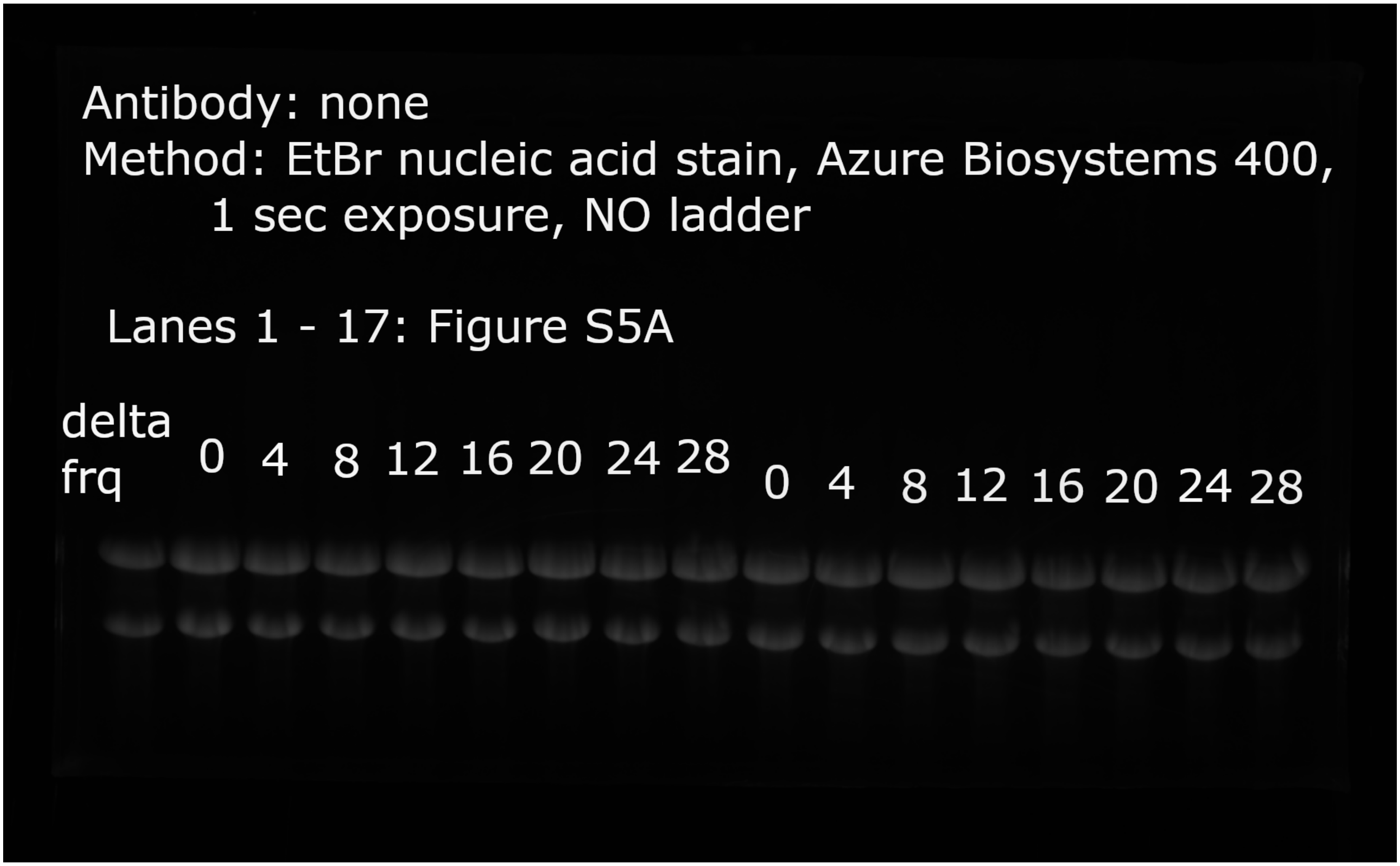
Antibody: none
Method: EtBr nucleic acid stain, Azure Biosystems 400, 1 sec exposure, NO ladder
Lanes 1 - 17: Figure S5A
delta frq
0 4 8 12 16 20 24 28 0 4 8 12 16 20 24 28
