# Supplementary material for: Nutritional compensation of the circadian clock is a conserved process influenced by gene expression regulation and mRNA stability
Source: PLoS Biol. 2023 Jan 5;21(1):e3001961. doi: 10.1371/journal.pbio.3001961 (PMC9848017; doi:10.1371/journal.pbio.3001961)
Supplement: Attachment — Submitted filename: ResponseToReviewers.docx [file pbio.3001961.s029.docx]

**Response to Reviewers (PBIOLOGY-D-22-01064R1)**

**Editor: “Reviewers 2 and 3 raise a number of important concerns which would need to be carefully and thoroughly addressed, with new data where needed, before we can consider your manuscript for publication. In particular, the reviewers have highlighted conclusions that are not yet adequately supported by the data, noting that additional work is needed to support the role of the identified candidate genes in nutritional compensation and your conclusion that this is a conserved process”**

We appreciate the insightful comments from the three independent Reviewers of this manuscript. We believe that the suggested experiments have greatly improved the mechanistic insights of this revised work on circadian Nutritional Compensation. Please see our full responses to Reviewers # 1, # 2, and # 3 below.

We note that updated Line numbers are provided in many of our responses below. Unfortunately, there is a known Microsoft Word software bug with Tracked Changes and inconsistent line numbering between pages (ex: <https://answers.microsoft.com/en-us/msoffice/forum/all/complaint-tracked-changes-causes-line-numbers-to/5d76fb35-8686-4e17-abe2-d85f2ef06b5e>). We have therefore marked the corresponding line number in the non-Tracked Changes version of the updated manuscript, with Tracked Changes Line numbers in parentheses.

**Reviewer 1 # 1: “Are these 16 factors also involved in the nutritional compensation besides glucose?”**

We appreciate the kind words from Reviewer # 1 in full support of this work. The minor question raised here is an excellent one—is the circadian clock normally compensated to other nutrients in addition to glucose (/ carbon sources) in fungi and in other circadian model systems? We believe this is a key point because fungi are often more nitrogen limited than carbon limited in their natural environment growing near or on plants. Nitrogen signaling is less studied in *Neurospora* compared to carbon / complex carbohydrate signaling, but recent high impact work has highlighted nitrogen sensing and signaling as a key gap in knowledge (ex. Huberman LB, et al. 2021. PNAS. PMID: 33753477). In preliminary experiments, we have begun to investigate the effects of amino acid source on circadian rhythms (see Figure below). The first author of the present study has recently accepted an Assistant Professor position, and this very question will be a major avenue of her future independent research.

**Figure Legend**: Circadian bioluminescence was recorded from race tube cultures of wild-type *Neurospora* grown on the indicated amino acids. Each amino acid was supplemented at 0.5% w/v, in the absence of a carbon source. Arginine serves as a high nutrient control (used throughout the revised manuscript), and the observed circadian period length is similar between high arginine (20.4 hours, black line) and high proline levels (20.9 hours, blue line). These data suggest that studying nitrogen compensation of the circadian clock in fungi would be a fruitful avenue for future research, building from historical data of circadian period length and rhythm robustness by race tubes containing different amino acids (Sargent ML, et al. 1972. Plant Physiology. PMID: 16658116).

**Reviewer 2:**

We appreciate the support from Reviewer # 2 of the “substantial work” presented in this study and thank this reviewer for their thorough comments and clarifying points. The manuscript has been manifestly improved by addressing each issue raised by the reviewer. Please note that Line numbers are provided for the revised manuscript as well as the revised manuscript in Microsoft Word with “Tracked Changes” in parentheses.

**# 1 [major]: “*Neurospora* ∆*set2* strain looks rhythmic to me (Fig. 5A) and period length is potentially not altered between low and high glc/arg levels […] 446-448: Is it completely arrhythmic? In supp Table 1 you write: 31.7+-0 // ARR. Does that mean it was rhythmic in one experiment? If it's not clear the experiment should be repeated with sufficient N's and data should be detrended to potentially uncover a masked rhythm […] 463-505 […] to my eye, ∆*set-2* oscillates at high nutrition levels with a similar period length as at low nutrition, albeit at low amplitude and potentially masked by the steeply increasing average bioluminescence […] data should also be detrended to reveal potential rhythms. This is important as the authors use the mammalian homolog for their cell culture experiments in the assumption that this is good candidate involved in NC”.**

We have now replicated the ∆*set-2* mutant 4 times in high nutrients compared to zero nutrients. The data below show each of the 4 high nutrient replicates side-by-side. We observe that the ∆*set-2* mutant is arrhythmic on high nutrients, and we note that this is completely consistent with a previously published finding (Figures 6D, 6E, and 6F from Zhao et al, 2013, PNAS, PMID: 24277852). We are in complete agreement that detrending is important for uncovering rhythms, especially as the amplitude of the signal increases over time; however, we do not observe any obvious or consistent rhythmic pattern between replicates for the ∆*set-2* mutant. For reader clarity, we have added a new Supplementary Movie # 3 showing one representative race tube from the data below.

We apologize for the confusing language in the original version of Supplementary Table 1 noting “31.7 hours / ARR” (arrhythmic) as the period length for ∆*set-2*. The “31.7 hours” was in reference to our period call algorithms, one of which searches for the best-fitting period in a range of ~16 – 32 hours. The algorithm reported “31.7 hours” as the closest-fitting period length to these arrhythmic signals. The revised table has been updated to read only “arrhythmic”.

**Figure Legend**: Circadian bioluminescence was recorded from race tube cultures of the Δ*set-2* mutant grown at high nutrients. N = 4 biological replicates are plotted individually, where vertical gray lines mark each 24-hour interval. There does not appear to be any consistent circadian signal between the replicates in either the background-corrected luminescence traces (top row) or in the traces where linear detrending has been applied (middle row). Linear detrending was also applied only to the positively-sloped region from hours 18 – 100 in the background-corrected traces, and there is no clear rhythmic pattern between the 4 replicates (bottom row).

**Reviewer 2 # 2 [major]: “Neither did ∆*cpsf6*, by the author's definition, have a large enough phenotype to be considered under-compensated. If the NC phenotype of ∆*cpsf6* is real but subtle, more N's are needed to show that the difference is statistically significant […] Moreover, *cspf6* is marked in the table but doesn't meet the authors' criteria for under-compensation […] 541-551 […] ∆*cpsf6* did not meet the threshold criteria the authors defined for mutants to be under-compensated. And the phenotype is only seen in old mycelia and in response to changes in arginine levels not glucose. So, it is hardly one of the most significant compensation phenotypes”**

We apologize for leaving the reviewer feeling we had over-stated the significance of the ∆*cpsf5* / ∆*cpsf6* knockout mutant hits in our genetic screen. To avoid this, language has been removed throughout the revised manuscript in reference to “top 2” screen hits. In response to the reviewer’s concern, we have reanalyzed our data and the steps that led us to study CPSF5 / CPSF6. We remain convinced that the phenotypes of the mutants are of interest.

Our logic for selecting ∆*cpsf5* / ∆*cpsf6* for further study begins on Line 362 (Tracked Changes Line 395). The primary and secondary genetic screen identified ∆*cpsf6* (NCU02152) as the most significant circadian period change among all the gene knockouts screened (with 4+ hours of period shortening). This discovery led us to informatically identify its putative binding partner in the CFIm complex and to examine the ∆*cpsf5* knockout mutant (NCU09014, marked as a “manual candidate” in Supplementary Table 1). Consistent with the model that CPSF5 and CPSF6 operate in a multimeric complex and that both subunits are required for function, each single mutant and the double mutant ∆*cpsf5* ∆*cpsf6* all show 3 – 4 hours shorter period length (Figure 4A, Supplementary Figure 5B, and Supplementary Movie 2).

The NC phenotype of ∆*cpsf5* / ∆*cpsf6* was then assessed for N = 6 – 7 biological replicates. The raw period length values are sorted and presented in the tables below:

| **Mutant** | **Media Type** | **Period Length (hr)** |  | **Mutant** | **Media Type** | **Period Length (hr)** |
| --- | --- | --- | --- | --- | --- | --- |
| ∆*cpsf5* | high nutrients (0.17% arginine) | 16.93220339 |  | ∆*cpsf6* | high nutrients (0.17% arginine) | 16.93220339 |
|  |  | 17.22413793 |  |  |  | 17.22413793 |
|  |  | 17.22413793 |  |  |  | 17.37391304 |
|  |  | 17.37391304 |  |  |  | 17.68141593 |
|  |  | 17.53171182 |  |  |  | 18.58966592 |
|  |  | 19.05622672 |  |  |  | 19.21864595 |
|  |  | 19.78217822 |  |  |  | 19.9879952 |
| ∆*cpsf5* | zero nutrients (0% arginine) | 19.02857143 |  | ∆*cpsf6* | zero nutrients (0% arginine) | 18.8490566 |
|  |  | 19.39805825 |  |  |  | 19.03029753 |
|  |  | 19.58823529 |  |  |  | 19.21153846 |
|  |  | 19.98 |  |  |  | 19.78217822 |
|  |  | 20.08496665 |  |  |  | 19.78217822 |
|  |  | 21.0525732 |  |  |  | 19.98 |
| **Average ± Standard Deviation Δ*cpsf5***: | | |  | **Average ± Standard Deviation Δ*cpsf6***: | | |
| 17.87 ± 1.09 hours high nutrients versus | | |  | 18.14 ± 1.15 hours high nutrients versus | | |
| 19.86 ± 0.7 hours zero nutrients (~2 hour  period change) | | | | 19.44 ± 0.47 hours zero nutrients (~1.3 hour period change) | | |

The wild-type *Neurospora* clock is slightly under-compensated (Figure 1); however, the average period length change between 0% glucose and 0.5% glucose was: 21.9 hours – 20.8 hours, or 1.1 hours. Positives in phase 1 and phase 2 of our screen were triggered by period differences > 1 hour (both ∆*cpsf5* and ∆*cpsf6* are well past this cutoff). In particular, the ∆*cpsf5* mutant (above, left) passed our criteria for period shortening with increasing nutrients in the tertiary phase of the screen as well.

**Reviewer 2 # 3 [major]: “Furthermore, period length of ∆*cpsf6*, which is short in general, is affected by arg concentration but not by glc concentration, while knockdown of its mammalian homolog in cell culture induces a long period phenotype, which is glc sensitive. It begs the question if the role in nutritional compensation of these to genes and the underlying mechanisms are indeed similar in fungi and mammals […] 553: In *Neurospora*, ∆*cpsf6* exhibited the NC phenotype in response to amino acid starvation and not glucose. Yet, in the cell culture experiments the knockdown of its mammalian homolog has a NC phenotype in response to changes in glucose levels. This is not discussed by the authors. It is also strange that knockout / knockdown of *cpsf6* both exhibit an under-compensated clock phenotype, yet has the opposite effect on period length in cell culture”**

We did not include the following data in the manuscript (original or revised) to avoid generating reader confusion about the ∆*cpsf5* / ∆*cpsf6* mutant phenotype. However, a combination of high glucose and high arginine causes the ∆*cpsf5* or ∆*cpsf6* knockout mutants to have a completely arrhythmic phenotype in the old mycelia region compared to controls:

Thus, we would argue that the ∆*cpsf5* / ∆*cpsf6* mutants are sensitive to glucose as well as arginine; however, the high arginine-depleted old tissue region revealed the very short period ~17 – 18 hour period length defect that was the most significant circadian period phenotype uncovered by our genetic screen.

To clarify the choice of investigating *CPSF6* over *CPSF5* knockdown in mammalian cell lines, this was due to reagent availability. We reached out to John Hogenesch and his expert lab manager Lauren Francey for guidance on gene knockdown assays in mammalian cells (see Acknowledgements). They generously provided protocols and recommended Qiagen’s “FlexiTube GeneSolution” siRNA. This Qiagen product comes with 4 different siRNA sequences targeting the same gene, each of which have been verified for accurate targeting and can be used at low individual concentrations. The use of 4 low concentration siRNAs lowers the toxicity and off-target effects of the knockdown procedure. Human *CPSF5* is not available in the 4x siRNA format (source: https://geneglobe.qiagen.com/us/product-groups/flexitube-sirna), while *CPSF6* is (# GS11052). Thus, we selected *CPSF6* for the gene knockdown experiments, reasoning that *CPSF5* and *CPSF6* are each essential members of the mammalian (and fungal) CFIm complex.

Lastly, we completely agree—it is puzzling that *CPSF6* knockdown in tissue culture lengthens circadian period, while ∆*cpsf6* significantly shortens period in *Neurospora*. We are confident in our *CPSF6* knockdown result given the preprint also observing period lengthening in *CPSF6* (Schmal et al, 2021, bioRxiv, https://doi.org/10.1101/2021.10.06.463236). Anecdotally, this is not the first time a period phenotype reversal has been observed between fungal/animal gene homologs with circadian relevance. The *prd-1* (NCU07839) mutant has a long period and nutritional over-compensation phenotype (Emerson et al, 2015, PNAS, PMID: 26647184). Its homolog in human, *DDX5* (reciprocal BLASTp E-value = 0), shortens period when knocked down (Padmanabhan et al, 2012, Science, PMID: 22767893). We speculate that effectors of compensation may be conserved through evolution, but their molecular targets may have diverged. This could be especially true in cases where the functional target is a core clock component not strongly conserved between species (e.g. FRH in *Neurospora*, TIM in flies, CRY in mammals, etc.).

**Reviewer 2 # 4 [major]: “the data gives direction as to where mechanisms for NC could be found It is a bit disappointing that rather than conducting follow up experiments that would help to pinpoint how the identified genes affect the clock machinery, such experiments are mostly announced to be done in “future work”. Data from published transcriptomes, as well as data from other publications, provide evidence that is partly circumstantial and partly contradictory. Potential connections are discussed but experiments showing or confirming a direct impact of the identified genes on NC of the clock are missing”**

We agree with the reviewer that the inclusion of more mechanistic follow-up experiments on Nutritional Compensation can only improve the work. To address this, we have improved the revised manuscript in 3 specific ways:

First, we moved the original Supplementary Figure 3 data, which described mechanistic details, into main text Figure 3. These data show that the *ck-1a*∆3’UTR mutant largely phenocopies the short period and nutritional under-compensation defect of knockout mutants lacking nonsense-mediated decay (NMD) machinery. We performed statistical tests as requested (see # 18 below) and find that the *ck-1a*∆3’UTR mutant phenotype is only slightly less severe than the NMD mutant ∆*upf1^prd-6^* (ANOVA p = 0.057, just under statistical significance). We then provide immunoblot evidence that WC-2 is ~1.5 – 2x more highly expressed in the ∆*upf1^prd-6^* background, thus rationalizing its slightly more severe under-compensation phenotype.

Second, we followed up on the ∆*set-1* long period and over-compensation defect and now provide more mechanistic details. ∆*set-1* was previously reported to be arrhythmic (Raduwan et al, 2013, Journal of Biological Chemistry, PMID: 23319591). To verify that the role of SET-1 in Nutritional Compensation involves its reported role as the catalytic subunit of the COMPASS complex, we investigated other COMPASS complex subunits. We discovered a second subunit of the complex, *swd3* (NCU03244), with a long period and over-compensation phenotype. To confirm that ∆*set-1* circadian defects are fully due to loss-of-function of the COMPASS complex, we generated a ∆*set-1* ∆*swd3* double mutant. The two single and double mutants show the same long period phenotype and similar compensation defects, indicating that SET-1, SWD3, and the COMPASS complex are required for normal Nutritional Compensation (please see new Supplementary Figure 7).

Lastly, we discovered that a mutant in *frh* can directly generate a nutritional over-compensation phenotype. Using a mutant with a second full-length copy of *frh* expressed at the *csr-1* locus, we characterized an over-compensation phenotype caused by doubling the expression of *frh*. Furthermore, we show that the second copy of FRH must have circadian function. We compared *frh* overexpression to a second mutant containing one copy of wild-type FRH and one copy of FRH^R806H^, an allele lacking circadian function. This FRH/ FRH^R806H^ mutant does not show the nutritional over-compensation phenotype. These data were added to the revised main text Figure 5. Taken together, these new results show that *ck-1a*, *wc-2*, and *frh* gene expression regulation are all important for maintaining Nutritional Compensation.

**Reviewer 2 # 5: “Lines 103-105: […] recent paper by Marzoll et al. (2022) showing that Neurospora FRQ as well as mammalian PER2 are phosphorylated by their respective CK1 in vitro on a circadian scale in a temperature compensated fashion […] should be cited here”**

This suggested citation has been incorporated at Line 104 (Tracked Changes Line 111).

**Reviewer 2 # 6: “120-122: While general transcription repressor RCO-1 has been shown to downregulate *frq* transcription as cited (Zhou et al. 2013), importantly, together with RCM-1 it is also part of the CSP-1 repressor complex (Sancar et al 2011). ∆*rco-1* has a more severe effect on rhythmic conidiation as well as period and amplitude of the clock than ∆*csp-1*, yet the metabolic compensation effect is not very different if one looks at the data presented in Olivares-Yanez et al, 2016. This should be considered and discussed also in the later sections where RCO-1 is mentioned (e.g. in the *pkac-1* mutant context).”**

This is an excellent point, and we are in complete agreement that both the ∆*csp-1* and ∆*rco-1* mutants have under-compensation phenotypes of similar magnitude (respectively: Sancar et al, 2012, Genes & Development, PMID: PMID: 23124067; Olivares-Yañez et al, 2016, Genetics, PMID: 27449058).

We have incorporated language at Line 126 (Tracked Changes Line 133; also referencing Sancar et al, 2011, Molecular Cell, PMID: 22152473) to include the model that the RCO-1 effect on Nutritional Compensation could be due to its repressive complex function with CSP-1.

**Reviewer 2 # 7: “Figure 2 […] what are the error bars of the bioluminescence traces if N=2 in almost all cases? It might be better showing both traces and the average”**

All error bars presented in this study represent standard deviations, as indicated in the Figure Legends throughout. A small subset of the *Neurospora* data presented in the main text do correspond to N = 2 biological replicates only (Figure 2B, Figure 3A), while a majority represent N ≥ 3 biological replicates (Figure 3C, Figure 4A, Figure 5A). Respectfully to this Reviewer, we believe that 4+ traces in every luciferase bioluminescence graph presented in this work would be much more difficult to read and interpret than the present data presentation.

**Reviewer 2 # 8: “Figure 2C: Please expand the abbreviation "FGSC" in the figure legend or in the text describing the figure”**

This change has been made in the Legends of: Figure 2, Figure 3, Supplementary Figure 2, and Supplementary Figure 3.

**Reviewer 2 # 9: “improve clarity [Figure 2C…] mention here what kind of experiments were performed in the individual screens, e.g. 1st screen: conidiation rhythms on glucose starvation race tubes, 2nd screen: luciferase reporter assays on 96-well plates -Glc -Arg…”**

This is a great suggestion and has been added to revised Figure 2C.

**Reviewer 2 # 10: “263ff and Supplementary Table 1 […] a little bit confusing with the number of the under-compensated mutants if you look at table S1. There, 14 genes are marked as under-compensated when mutated. In line 263 the authors examine "the group of 12 under-compensated mutants". I know the other two are looked at more closely from 311ff but this is still confusing. Please rewrite for clarity”**

We apologize for the confusing mutant numbering system in the original manuscript. The revised Supplementary Table 1 marks 13 total genes as nutritional under-compensation mutants, including 1 positive control with a published phenotype, ∆*csp-1* (Sancar et al, 2012, Genes & Development, PMID: 23124067). The 12 novel under-compensation mutants identified by our genetic screen should now be clearly derived from Supplementary Table 1 and numbered correctly throughout the main text.

**Reviewer 2 # 11: “Please mention that ∆*upf1* and ∆*upf2* are the two most significant hits in 265. Otherwise they would not be mentioned at all in the main text”**

We appreciate this clarification, which has been incorporated starting at Line 284 (Tracked Changes Line 298).

**Reviewer 2 # 12: “Supplementary Table 1: *mek-1* is listed in the table as RNA binding and not as a (MAPK-)kinase”**

MEK-1 (NCU06419) is now correctly listed as a kinase in revised Supplementary Table 1 as well as revised Figure 2C.

**Reviewer 2 # 13: “Supplementary Table 1: […] Please write “Arrhythmic” instead of ARR”**

This is now corrected in revised Supplementary Table 1 in two locations.

**Reviewer 2 # 14: “Supplementary Table 1: […] Define “output” in primary screen”**

This has been clarified in the revised Supplementary Table 1 Legend and in the main text at Line 225 (Tracked Changes Line 235).

**Reviewer 2 # 15: “Supplementary Table 2: The table is used to show potential clock or light regulation of the screen results. Therefore, for easier reading, only the potential genes involved in temperature [/nutritional] compensation should be shown or at most the 52 strains plus controls from the tertiary screen”**

Revised Supplementary Table 2 contains 2 tabs: the first tab (“tertiary_screen_genes”) lists only the gene knockouts that progressed to the tertiary screen, and the second tab (“all_screen_genes”) lists all NCUs screened (matching original Table S2). The revised Supplementary Table 2 Legend has been updated accordingly.

**Reviewer 2 # 16: “277-279: I cannot follow the reasoning why the other under-compensation mutants were not pursued further as they do meet the period ratio criteria”**

In the revised text, we highlight 6 of 12 novel under-compensation mutants in Figure 3. Beginning at Line 289 (Tracked Changes Line 303), we have clarified that the remaining 6 under-compensation mutants were discovered but not pursued further mechanistically. The data obtained from those 6 knockout mutants were moved to Supplementary Figure 3 to create space for mechanistic data on the nonsense-mediated decay (NMD) under-compensation phenotype.

**Reviewer 2 # 17: “Figure 3: The *prd-4* (S493L) mutant does not really belong here and the authors should consider taking it out completely as, in contrast to the other strains tested, it is a gain of function mutant. The Dunlap/Loros labs published quite some time ago that the mutation enhances interaction of PRD-4 with FRQ leading to phosphorylation of FRQ by this checkpoint kinase thereby accelerating the clock (Pregueiro et al, 2006). The *prd-4* KO strain did not show altered nutritional compensation (see Supplementary Table 1). With the space gained the bar graph could be enlarged a little bit”**

We are in complete agreement that the classical allele *prd-4* has a very different phenotype from the ∆*prd-4* knockout mutant, as indeed we showed in the 2006 Science article. As described starting from Line 219 (Tracked Changes Line 229), all of the classical *prd* alleles were included in our genetic screen, and the ∆*prd-4* knockout mutant was included as part of the kinase knockout collection library. Indeed, our data suggest that the gain of function *prd-4* mutant may alter Nutritional Compensation, while the knockout has no effect (Supplementary Table 1). This same phenomenon was observed in 2006 for circadian period change and for Temperature Compensation.

A subset of the original version of Figure 3, including the *prd-4* data, was moved to Supplementary Figure 3. This did generate space to increase the bar graph size in the revised Figure 3.

**Reviewer 2 # 18: “299: It is very interesting that the CK1a 3'UTR mutant has a similar NC phenotype as ∆*upf-1^prd-6^*. To state that the phenotype of the *ck1a* gene mutant is “not as severe” as the phenotype of ∆*upf-1^prd-6^*, though, would require statistical analysis of a sufficiently large set of independent experiments, as this would be a rather important point the authors make here”**

We agree that the *ck-1a*∆3’UTR mutant phenotype (short period and under-compensated) is very interesting, and have moved this exciting result into the main text Figure 3.

This is an excellent suggestion to substantiate our claim that the *ck-1a*∆3’UTR mutant phenotype is less severe than the ∆*upf1^prd-6^* knockout (i.e. the nutritional under-compensation defect is 1-hour decreased in magnitude, see Revised Figure 3C). The biological replicate sample sizes are as follows: *ras-1^bd^* control: N = 4 biological replicates for zero nutrients, N = 4 for high nutrients; *ras-1^bd^* ∆*upf1^prd-6^*: N = 3 for zero nutrients, N = 3 for high nutrients; *ras-1^bd^* *ck-1a*∆3’UTR: N = 3 for zero nutrients, N = 3 for high nutrients.

Using these biological replicates, we performed an ANOVA and Tukey HSD test for the zero nutrient period length data, and for the high nutrient period data. Our statistical question was: is the circadian period length significantly affected by strain genotype across the same nutrient conditions? Below we have included screenshots of our statistical tests performed in R:

We draw two main conclusions from these statistical tests: first, the *ck-1a*∆3’UTR and ∆*upf1^prd-6^* mutants have significantly shorter period length than the control in both nutrient regimes tested. Second, the p-values are ~4x lower and more significant for ∆*upf1^prd-6^*, to the extent that the *ck-1a*∆3’UTR and ∆*upf1^prd-6^* period difference nearly reaches significance (p = 0.057) under high nutrients. We have incorporated this analysis into the revised Figure 3 Legend as well as the main text at Line 343 (Tracked Changes Line 376). We also removed any language that ∆*upf1^prd-6^* has a *significantly* more severe under-compensation phenotype than *ck-1a*∆3’UTR, but we note the ANOVA p-value is very close to p = 0.05. The newly added WC-2 overexpression data strongly indicate that the 1 additional hour of period shortening in ∆*upf1^prd-6^* is caused by both CKIa and WC-2 gene expression regulation, where the majority of the period length and compensation effect is due to CKIa regulation.

**Reviewer 2 # 19: “301: Figure 5B doesn't show this! Are *wc-2* and *frh* both up-regulated in the NMD mutants?”**

The reviewer is referring to the statement, “NMD mutants also show ~1.4-fold changes in *wc-2* and *frh* gene expression levels (Kelliher et al., 2020a: Figure 5B; Wu et al., 2017)”. We apologize for the unclear language—the original text was referring to Figure 5B from Kelliher et al, 2020, eLife, PMID: 33295874, not Figure 5 of the present study. Revised text Line 349 (Tracked Changes Line 382) now clarifies that *wc-2* is up-regulated and *frh* is down-regulated in NMD mutants.

**Reviewer 2 # 20: “301-310: Here, western blots and qRT-PCR data should be shown, confirming data from transcriptome analyses cited that in the mutants *wc-2* and *frh* transcript as well as the respective protein levels are altered”**

We appreciate this excellent point, and have incorporated strong protein abundance data into the revised Figure 3 (part D) showing: 1) CKIa levels are highly upregulated in both the *ck-1a*∆3’UTR and ∆*upf1^prd-6^* mutant backgrounds (∆*upf1^prd-6^* finding completely consistent with our previously published results: Kelliher et al, 2020, eLife, PMID: 33295874); 2) WC-2 levels are ~1.7-fold upregulated in the ∆*upf1^prd-6^* mutant, consistent with the RNA-Seq result; and 3) FRH levels are unchanged in the ∆*upf1^prd-6^* mutant, inconsistent with the RNA-Seq result. Interestingly, this hints at translational-level regulation of FRH, which may be interesting to pursue in future work.

**Reviewer 2 # 21: “312: “compared to the 11 remaining under-compensation mutants”. 11 in this case because the authors probably did not include the *csp-1* “positive control” strain?! Please be consistent with the numbers”**

As with comment # 10 above, this has been corrected to read 12 total novel strains with under-compensation defects, not including the 1 positive control mutant with a published phenotype ∆*csp-1* (revised Supplementary Table 1).

**Reviewer 2 # 22: “365-372: For comparison to Figure 4B it would be good to show representative examples for the 3 groups of changes in the APA landscape in the supplementary section”**

This is an excellent suggestion to further display the large changes in the APA landscape in the mutant. A new Supplementary Figure 6 has been added to show 4 representative examples of the alternative polyadenylation (APA) alterations observed in the ∆*cpsf5* ∆*cpsf6* double mutant.

**Reviewer 2 # 23: “378-429, Figure 4: In this screen only two (of the handpicked) genes had altered APA in the double mutant. Apparently, however, this did not change gene expression levels of these genes. On the other hand, genes with altered expression levels in the double mutant did not exhibit an altered APA landscape in the assay. So, there is no correlation and thus no indication that control of APA has a direct effect on NC. Is there an effect on protein translation levels maybe? Additionally, altered gene expression levels should have been confirmed via qRT-PCR”**

Figure 4 was not meant to show that “control of APA has a direct effect on NC”, but meant to show that loss genes resulting in a Nutritional Compensation phenotype, and associated with APA (∆*cpsf5* ∆*cpsf6),* did indeed result in alterations APA; this is plainly the case. We had hoped to also find the actual targets of APA involved, and we agree that the APA data as presented in Figure 4 do not present a single, clear downstream candidate gene for the short period length nor Nutritional Compensation phenotype of ∆*cpsf5* ∆*cpsf6* mutants. This was somewhat disappointing, although it was also a long shot given the genome-wide analysis presented in Figure 4. Furthermore, ∆CFIm altered hundreds of genes and ~50% of the APA landscape. It is possible that a single clear candidate gene could emerge, but this would require a careful examination of all 471 gene knockouts with altered APA (a screen already larger than the one presented in this study). It is also possible that multiple CFIm target genes with minor circadian compensation effects are all responsible for the observed ∆*cpsf5* ∆*cpsf6* phenotype.

We understand the reviewer’s urge to suggest confirmation using RT-qPCR, but wish to mention that the need to validate RNA-seq data is being widely re-examined (e.g. Everaert C, et al. 2017. Scientific Reports. PMID: 28484260; Coenye T. 2021. Biofilm. PMID: 33665610). Inconsistencies between gene expression levels reported by microarrays and RT-qPCR were not uncommon, but with the emergence of the superior RNA-seq technologies, inconsistencies between RNA-seq and RT-qPCR (especially biological replicates of RNA-seq data examining mid to highly expressed genes) are quite rare. Additionally, no conclusions regarding NC are ascribed to the reported expression differences in Figure 4. To answer the question: is *ckb-1* (NCU05485) expression affected at the translational level in ∆*cpsf5* / ∆*cpsf6* mutants compared to controls, we had hoped to use a CK2β1 antibody from previous work (Figure 3 from Mehra et al, 2009, Cell, PMID: 19450520). Unfortunately, this antibody was lost to time in the freezers.

**Reviewer 2 # 24: “438-439: briefly mention how PKA regulates RCO-1 (inactivation of the repressor by phosphorylation). Also see above regarding RCO-1. Observation is also compatible with inhibition of CSP-1/RCO-1/RCM-1 complex.”**

Please see comment # 6 above, where we have added a citation to Sancar et al, 2011, Molecular Cell (PMID: 22152473) to include the model possibility that the RCO-1 effect on Nutritional Compensation could be due to its repressive complex function together with CSP-1.

While we agree that the ∆*rco-1* and ∆*csp-1* under-compensation phenotypes are consistent with each other (~2-hour period shortening across nutrients), the ∆*pkac-1* phenotype is in the opposite direction (slight extended compensation) and ∆*rcm-1* appears to be dispensable for normal compensation. The wild-type clock has an average under-compensation of 1.1 hours (see comment # 2 above for full calculation), thus ∆*rco-1* and ∆*csp-1* are each ~1 additional hour shorter than wild-type and ∆*pkac-1* is ~1 additional hour longer than wild-type. Therefore, the model does not seem quite as straightforward as PKA activates RCO-1/RCM-1 to prevent WCC-independent *frq* transcription (∆*pkac-1*, ∆*rcm-1*, and ∆*rco-1* phenotypes should match) or PKA activates CSP-1/RCO-1/RCM-1 to prevent WCC overexpression (∆*pkac-1* phenotype should match ∆*csp-1*). We have added text to clarify an additional model of PKA directly regulating WCC and/or FRQ in a nutrient-dependent manner. We are not aware of any knockout, PKA target analysis, or other datasets showing that PKA phosphorylates and inactivates RCO-1, only its positive regulation of RCM-1 as cited at Line 490 (Tracked Changes Line 543). We apologize if we have missed this key piece of prior literature evidence in our current model for Nutritional Compensation mechanisms.

**Reviewer 2 # 25: “471: “Δ*set-2* mutant results in … high *frq* expression levels … (Zhou et al., 2013; Sun et al., 2016).” This observation is not reproduced in the ∆*set-2* transcriptome dataset from Bicocca et al 2016 shown in Figure 5C. Curiously FRQ protein levels in ∆*set-2* were not shown to be elevated in Sun et al., 2016. They were actually slightly lower. The observed decrease in *frh* found by Bicocca could account for this but these data would need to be confirmed via qRT-PCR or western blots with the strains used in this work”**

We concur that the previously published molecular data for the ∆*set-2* mutant are peculiar with high *frq* mRNA levels (consistent with increased WCC-independent *frq* transcription) but a ~30-40% reduction in FRQ protein levels (not consistent with higher *frq* mRNA abundance). It should be noted that the Δ*set-2* RNA-seq data from Bicocca et al were generated in the Selker lab, who have resisted efforts to consider or control for either light or circadian issues during experiments. In the case of Bicocca et al, the data were generated from cultures grown in unknown light conditions at 32°C, a temperature at which the high temperature-driven *frq*/FRQ expression might obscure other effects. In mining this dataset, we believed that differences in expression of *frh,* which is not reported to be temperature- or light- dependent like *frq,* ought to be reliable compared to controls done under the same conditions.

Please see comment # 4 above, where we demonstrate directly that *frh* gene expression is involved in Nutritional Compensation, and also comment # 26 below, where we have successfully shown that WCC-independent *frq* transcription is likely nutrient-dependent. It may be that the ∆*set-2* arrhythmicity on high nutrients is explained by constitutive and high WCC-independent *frq* transcription. The ∆*set-2* low amplitude and short period clock on zero nutrients is not yet mechanistically defined, but our findings relating *frh* gene expression to over-compensation is a potential explanation.

**Reviewer 2 # 26:** “**472-474: The authors should back their claim and show that WCC-independent *frq* transcription is indeed nutrient dependent using the appropriate strains”**

We thank the reviewer for this suggestion—please see new data provided in Figure 4D. As mentioned above in comment # 25, WCC-independent *frq* transcription appears to be low in zero nutrients and higher in nutrient replete conditions. This finding rationalizes the ∆*set-2* arrhythmic phenotype on high nutrients and was an excellent suggestion.

**Reviewer 2 # 27: “The most convincing, interesting and obvious candidates to investigate NC in cell culture would have been *upf-1* and *upf-2* as well as *set-1* or *prd-1*. Why not analyze homologs of these? So, while the authors did find an NC phenotype in the siRNA knockdown of the homolog genes, the reasoning behind picking these genes leaves me puzzled”**

We apologize if the logic presented in the original study justifying our choice of *CPSF6* and *SETD2* for follow-up study in a mammalian circadian system was unclear. Of course, we searched for evolutionarily related homologs among all of the novel hits from our Nutritional Compensation genetic screen. We then investigated each homologous gene’s knockdown circadian profile published in Zhang et al, 2009, Cell (PMID: 19765810) and available at the following database: http://biogps.org/ (under the “Circadian Genomics Screen” plugin). We selected *CPSF6* and *SETD2* based on 1 out of 2 promising results from the genome-wide siRNA screen (please see screenshots below). Additional text has been added at Line 656 (Tracked Changes Line 725) to clarify our selection of *CPSF6* and *SETD2*:

*SETD2* knockdown in 2009 U2OS screen:

*CPSF6* (left) and *CPSF5* (right) knockdown in 2009 U2OS screen:

The *prd-1* (NCU07839) homolog *DDX5* (and its paralog *DDX17*) already has a published short period phenotype (Padmanabhan et al, 2012, Science, PMID: 22767893), and thus we selected different genes to increase the impact of this present study.

The *set-1* (NCU01206), *upf1* (NCU04242), and *upf2* (NCU05267) mammalian homologs would certainly be of interest to follow in future work. However, the *UPF2* knockdown dataset from U2OS cells appears to show a consistently wild-type circadian period length:

**Reviewer 2 # 28: “Figure 6: Are the differences in period length statistically significant for CPSF1 and SETD2 in 6A?”**

No, none of the period length differences in Figure 6A are statistically significant by Student’s t-test, although the *CPSF6* knockdown is approaching significance, and the p-values are much lower in the *CPSF6* and *SETD2* knockdown conditions compared to each control (*CRY2* and siRNA negative):

Negative control: low glucose average period = 24.06 hours, high glucose average = 23.94 hours, p = 0.73

*CRY2*: low glucose average = 28.14 hours, high glucose average = 28.06 hours, p = 0.94

*CPSF6*: low glucose average = 27.19 hours, high glucose average = 25.62 hours, p = 0.08

*SETD2*: low glucose average = 25.18 hours, high glucose average = 26.54 hours, p = 0.24

As is apparent in Figure 6A, knockdown of these genes severely affected the robustness of the rhythms under low glucose conditions. 4 – 5 cycles were reliably achieved with negative siRNA controls, whereas low glucose rhythms were often lost after 2 – 3 cycles in gene-specific knockdowns. It is possible that the *CPSF6* knockdown under-compensation phenotype would have achieved statistical significance if the rhythms could have been followed for more cycles (e.g. see Figure 6B for *SETD2*, a novel circadian and compensation phenotype).

**Reviewer 2 # 29: “What are the error bars in the Bioluminescence traces in 6A?”**

Bioluminescence error bars in Figure 6A are ± 1 SD reflecting technical and biological replicate traces averaged together, as with *Neurospora* data presented throughout the manuscript. All Figure Legends now indicate standard deviation error bars as well as the Methods section at Line 1094 (Tracked Changes Line 1169).

**Reviewer 2 # 30:** “**563-564: looks more like the luciferase expression levels are higher than the amplitude of oscillation. See/compare also Fig 6B”**

The *SETD2* knockdown traces show both increased magnitude (i.e. values on the Y-axis) and increased amplitude compared to the respective control. Plotting siRNA negative control curves on the same Y-axis scaling as *SETD2*:

**Figure 6A** subset, re-plotted

**Figure 6B** subset, re-plotted

Taking the high glucose (yellow) traces from Figure 6B above as a sample amplitude calculation and using the formula [Amplitude = (Max value – Min value) / 2]:

- control peak 1 = 940.0 units (at 17 HPS); control trough 1 = 440.5 units (at 31 HPS); control peak 2 = 637.2 units (at 41 HPS); control trough 2 = 361.3 units (at 55 HPS); control amplitudes = 249.75 and 137.95 units
- *SETD2* peak 1 = 1913.5 units (at 18 HPS); *SETD2* trough 1 = 915.5 units (at 33 HPS); *SETD2* peak 2 = 1341.7 units (at 42 HPS); *SETD2* trough 2 = 532.8 units (at 57 HPS); *SETD2* amplitudes = 499 and 404.45 units

**Reviewer 2 # 31: “781-782: format reference”**

We appreciate the identification of this error, and this has been fixed at Line 902 (Tracked Changes Line 975).

**Reviewer 3 # 1: “characterization of these regulatory factors [of nutritional compensation in the circadian clock] are lacking or very limited”**

We appreciate the kind words from Reviewer # 3 in support of the novelty, broad appeal, and promise of this work, and agree that modification of the manuscript to provide additional mechanistic detail on the regulators of Nutritional Compensation identified through our fungal genetic screen could only add value to or work. As noted in responses to Reviewer # 2, we have improved the mechanistic insights of the revised manuscript in 3 specific ways:

First, we moved the original Supplementary Figure 3 data (which described mechanistic details) into main text Figure 3. These data show that the *ck-1a*∆3’UTR mutant largely phenocopies the short period and nutritional under-compensation defect of knockout mutants lacking nonsense-mediated decay (NMD) machinery. We then provide immunoblot evidence that WC-2 is ~1.5 – 2x more highly expressed in the ∆*upf1^prd-6^* background, thus rationalizing its slightly more severe under-compensation phenotype compared to the novel *ck-1a*∆3’UTR mutant.

Second, we followed up on the ∆*set-1* long period and over-compensation defect and now provide more mechanistic details. ∆*set-1* was previously reported to be arrhythmic (Raduwan et al, 2013, Journal of Biological Chemistry, PMID: 23319591). To verify that the role of SET-1 in Nutritional Compensation involves its reported role as the catalytic subunit of the COMPASS complex, we investigated other COMPASS complex subunits. We discovered a second subunit of the complex, *swd3* (NCU03244), with a long period and over-compensation phenotype. To confirm that ∆*set-1* circadian defects are fully due to loss-of-function of the COMPASS complex, we generated a ∆*set-1* ∆*swd3* double mutant. The two single and double mutants show the same long period phenotype and similar compensation defects, indicating that SET-1, SWD3, and the COMPASS complex are required for normal Nutritional Compensation (please see new Supplementary Figure 7).

Lastly, and most interestingly, we discovered that a mutant in *frh* can directly generate a nutritional over-compensation phenotype. Using a mutant with a second full-length copy of *frh* expressed at the *csr-1* locus, we characterized an over-compensation phenotype caused by doubling the expression of *frh*. Furthermore, we show that the second copy of FRH must have circadian function. We compared *frh* overexpression to a second mutant containing one copy of wild-type FRH and one copy of FRH^R806H^, an allele lacking circadian function. This FRH/ FRH^R806H^ mutant does not show the nutritional over-compensation phenotype. These data were added to the revised main text Figure 5. Taken together, these new results show that *ck-1a*, *wc-2*, and *frh* gene expression regulation are all important for maintaining Nutritional Compensation, further supporting our model that multiple mechanisms at the transcriptional and post-transcriptional levels underlie circadian compensation to nutrients.

**Reviewer 3 # 2: “data quality for CFIm-mediated regulation of mRNA alternative polyadenylation seemed poor […] no data is provided to suggest that CFIm-mediated regulation of mRNA 3' UTRs is involved in nutritional compensation”**

∆*cpsf6* had the shortest period phenotype observed in the entire primary and secondary screens (Supplementary Table 1), so its role in period control under nutrient starvation was established and of plain interest. In Figure 4, we aimed to confirm that loss of these genes did in fact affect APA, to describe the extent and magnitude of this effect, and to identify candidate transcripts whose UTRs were affected and that might be involved in Nutritional Compensation. We demonstrated widespread APA in *Neurospora*, and showed that roughly 50% of the APA events in the transcriptome are affected by loss of CFIm, but screening of all 471 of these was not feasible. To our disappointment, we could not identify a clear downstream candidate gene for the short period length nor Nutritional Compensation phenotype of ∆*cpsf5* ∆*cpsf6* mutants. There could be many reasons for this: APA might affect translation efficiency rather than only RNA expression of the relevant gene(s), as was recently demonstrated of the mammalian CFIm complex and its circadian function (Schmal C, et al. 2021. bioRxiv. <https://doi.org/10.1101/2021.10.06.463236>). However this disappointment does not diminish the anchoring observations that ∆CFIm impacts Nutritional Compensation and, as expected, ∆CFIm impacts APA.
