# Supplementary material for: Nutritional compensation of the circadian clock is a conserved process influenced by gene expression regulation and mRNA stability
Source: PLoS Biol. 2023 Jan 5;21(1):e3001961. doi: 10.1371/journal.pbio.3001961 (PMC9848017; doi:10.1371/journal.pbio.3001961)
Supplement: Attachment — Submitted filename: ResponseToReviewers.docx [file pbio.3001961.s030.docx]

**Response to Reviewers (PBIOLOGY-D-22-01064R2)**

**Editor: “the reviewers are largely satisfied with the revision, although Reviewer 2 has raised two minor concerns. Therefore, […] satisfactorily address the remaining points raised by Reviewer 2.”**

We appreciate the comments from the three original Reviewers of this manuscript. Please see our full responses to Reviewer # 2 below.

**Reviewer 2:**

**Reviewer 2 # 1: “Figure 3: The figure legend needs to be adjusted to the new figure. Please check.”**

We appreciate this reminder and have thoroughly checked all main text and Supplementary figure legends for consistency with figure content.

**Reviewer 2 # 2: “regarding PKA and RCO-1/RCM-1: In my original comment I asked to briefly mention how PKA regulates RCO-1. That was an error on my part. I meant to ask the authors to mention how PKA regulates RCO-1/RCM-1. However, the authors are mistaken that "PKA activates RCO-1/RCM-1...". The opposite is the case: Liu et al. 2015 found that "PKA regulates *frq* transcription by inhibiting RCM-1 activity through RCM-1 phosphorylation", which is good because this is compatible with their other results. Please check and revise.”**

This reviewer is absolutely correct, and we sincerely apologize for our mistake in the previous versions of the text. This mistake has been corrected at Line 496 (Tracked Changes Line 499), where it now correctly states that PKA inhibits RCM-1 by phosphorylation, thus activating WCC-independent *frq* transcription (PKA --| RCM-1/RCO-1 --| WCC-independent *frq* transcription).

We agree that our data is consistent with the model that WCC-independent *frq* transcription plays a role in nutritional compensation: ∆*rco-1* and ∆*csp-1* show ~1 hour under-compensation compared to wild-type (and WCC-independent *frq* transcription should be activated), while the ∆*pkac-1* phenotype is in the opposite direction of slight extended compensation by ~1 hour compared to wild-type (and WCC-independent *frq* transcription should be inhibited).
